# Ductility Variation and Improvement of Strain-Hardening Cementitious Composites in Structural Utilization

**DOI:** 10.3390/ma17040831

**Published:** 2024-02-08

**Authors:** Pinxin Diao, Zongyou Ling, Yunbo Bai, Weihua Lu, Yongxing Zhang

**Affiliations:** 1 School of Civil Engineering, Nanjing Forestry University, Nanjing 210037, China; 2 Industry and Information Technology Bureau of Fangchenggang City, Fangchenggang 538001, China; 3 Shanghai Civil Engineering Group Co., Ltd., of CREC, Shanghai 200436, China

**Keywords:** crack elongation performance, ductility variation and improvement, strain hardening cementitious composite, structural utilization, steel reinforcement, experimental investigation

## Abstract

Strain-hardening cementitious composite (SHCC) has the obvious advantages of excellent material properties such as its high tensile and compressive strengths, high tensile strain capacity, and excellent durability against multi-cracking performance with very fine crack widths. In particular, the multi-cracking performance of SHCC during structural utilization is obviously reduced compared to that of SHCC in uniaxial tension tests using dumbbell-shaped specimens of small size. The corresponding tensile strain capacity of SHCC during structural utilization is, thus, significantly decreased compared to that of SHCC in uniaxial tension tests. However, the reduction in the ductility of SHCC during structural utilization has not been sufficiently understood, and further study is required. This paper presents an experimental investigation into the ductility variation of flexural-failed and shear-failed SHCC members as well as the ductility improvement of SHCC members with steel reinforcement compared with that of SHCC in uniaxial tension tests using small-sized specimens. This study focuses on not only the decrease in the crack elongation performance of the SHCC material during structural utilization but also the increase in the crack elongation performance of SHCC members with steel reinforcement. The results demonstrate that the crack elongation performance of flexural-failed and shear-failed SHCC members is significantly reduced compared to that of SHCC in the uniaxial tension tests. Moreover, it was confirmed that steel reinforcement can effectively improve the SHCC member, increasing the strain-hardening capacity and multi-cracking performance. The load-carrying capacity of the flexural-failed SHCC member with steel reinforcement seemed to increase linearly with an increase in the reinforcement ratio, accompanied by an increase in the distribution of multiple fine cracks in the flexural-failed SHCC member with steel reinforcement.

## 1. Introduction

Many concrete structures that have not yet reached their service life have suffered damage in the last few decades, mainly induced by deficiencies in concrete brittleness and the disadvantages of concrete when resisting tensile forces [1,2,3]. In view of previous research, many different cementitious composites have been developed as construction materials to overcome the aforementioned deficiencies of concrete, although they also have obvious shortcomings in repairing and rehabilitation works, for which fiber-reinforced concrete (FRC) has the disadvantages of quasi-brittleness and insufficient ductility [4,5,6,7]. Therefore, improving the strength and strain capacity of cementitious composites has been a big issue in the development of high-performance construction materials.

Compared with common building materials, strain-hardening cementitious composite (SHCC) is an absorbing building material with the well-known advantage of having significant strain-hardening properties [8,9], which correlates with its multi-cracking performance dominated by the fiber bridging effect resulting from the matching between the fibers and matrix in the SHCC material [10,11].

In recent years, SHCC has been increasingly used in structural projects given its notable advantages of not only having high tensile and compressive strengths but also its aforementioned high tensile strain capacity and excellent durability and multi-cracking performance, with very fine crack widths [12,13,14,15]. In particular, many published works have demonstrated that SHCC has a great capacity to resist shear failure in comparison to normal concrete and fiber-reinforced concrete [8]; SHCC exhibits an outstanding ability to strengthen concrete members subjected to seismic and shear action [16].

Many studies have examined the application of SHCC materials for structural use; however, the crack elongation performance of SHCC utilized in large structures is obviously reduced compared to that of SHCC in uniaxial tension tests using dumbbell-shaped specimens of small size. This is because the bridging effect of the short fibers in the matrix of SHCC can significantly vary; the fiber bridging effect in SHCC specimens subject to the uniaxial tension test is correlated with all the nearby fibers and, thus, continuously changes in the SHCC member [17]. Moreover, the known reduction in the crack elongation performance of SHCC when used in large structures is dominated by the aforementioned fiber bridging effect [11], which is difficult to clarify due to the complex influences of not only the fiber distribution within one section of SHCC material but also the variation in fiber content among different sections. Indeed, the available literature on these influences remains limited [18]. There is, therefore, an urgent to investigate the variation in the ductility of SHCC materials and improve their use in large-scale structural projects.

This paper presents an experimental investigation into the ductility variation of shear-failed and flexural-failed SHCC members as well as the improvement in the ductility of SHCC members with steel reinforcement compared with that of SHCC subject to the uniaxial tension test using dumbbell-shaped specimens of small size. This study focuses on not only the decrease in the crack elongation performance of SHCC during structural utilization but also the improvement in its ductility. Thus, this study indicates that employing steel reinforcement can increase the crack elongation performance of SHCC when used in large structural projects.

## 2. Materials and Methods

### 2.1. Mix Design of the SHCC Material

Table 1 shows the mix design of the SHCC material used in the experiment. The mix design had a water:binder ratio of 0.22, in which the binder comprised silica fume and low-heat Portland cement. As listed in Table 1, 15% of the cement content was replaced by the silica fume, and quartz sand was employed as the fine aggregate in the mix design. Polyethylene (PE) fibers with high strength were adopted in the SHCC material, with a volume of 1.5%, which were 0.012 mm in diameter and 6 mm in length. Moreover, a superplasticizer was adopted to improve the matrix workability of the SHCC material.

### 2.2. Specimen Characteristics

#### 2.2.1. Dumbbell-Shaped SHCC Specimens for the Uniaxial Tension Tests 

**Figure 1:**
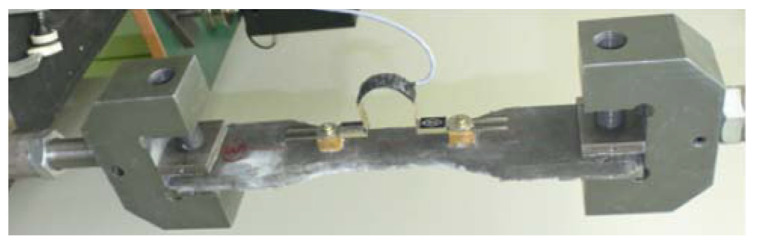
Dumbbell-shaped specimen of the SHCC material in the uniaxial tension test.

Figure 1 shows a specimen of the SHCC material and the setup of the uniaxial tension test, in which the small-sized specimen with a dumbbell shape had a 13 mm × 30 mm cross-section. In order to measure the opening displacement of the small-sized specimen after cracking, displacement transducers were bonded to both surfaces of the small-sized specimen. The specimen with a dumbbell shape had a length of 100 mm and, thus, the strain of the SHCC material was the percentage elongation of the 100 mm specimen. The transducer had a ± 5 mm gauge capacity, 100 mm gauge length, and 1/2000 mm sensitivity. The load was applied at a loading rate of 0.2 mm/min and measured using the load cell with a 50 kN gauge capacity. Three cylindrical specimens with a 50 mm diameter and 100 mm length were employed in the uniaxial compression tests, for which the specimens were cured for 28 days.

#### 2.2.2. Specimens of the Shear-Failed SHCC Member

**Figure 2:**
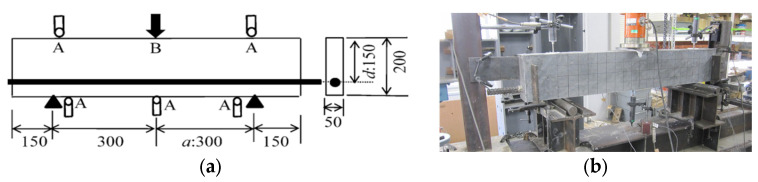
Geometry and setup of the experimental SHCC specimen during shear failure. (**a**) Geometry of the specimen (unit: mm); (**b**) Experimental setup.

Figure 2 demonstrates the geometry and setup of the experimental SHCC specimen in the shear failure test. The prepared SHCC member had a length of 1200 mm and a cross-section of 50 mm × 200 mm; the specimen had a width of 50 mm and an effective depth of 150 mm. The specimen had a shear span length of 300 mm where a/d = 2, and the longitudinal steel reinforcement was a 25 mm diameter deformed steel bar with an elastic modulus of 200 GPa and a yield strength of 1050 MPa. This was expected to avoid the sudden fracture of the shear-failed SHCC member during the experiment due to the high tensile strength of the deformed steel bar. In the experiment, the loading of the specimen was implemented using a three-point bending test setup, in which displacement was measured at the points labeled “A” in Figure 2 and the load was measured at point “B”. The load was measured using a load cell with a 300 kN gauge capacity and sensitivity of 0.1 kN, and the transducer used to measure the displacement had a 25 mm gauge length and a sensitivity of 0.002 mm. The transducer used to measure the displacement required adjustment during the testing process when the measured displacement was greater than the 25 mm gauge range of the displacement transducer. The experiment was ended when a sudden drop occurred in the curve of load versus displacement.

#### 2.2.3. Specimens of the Flexural-Failed SHCC Member with and without Steel Reinforcement

**Figure 3:**
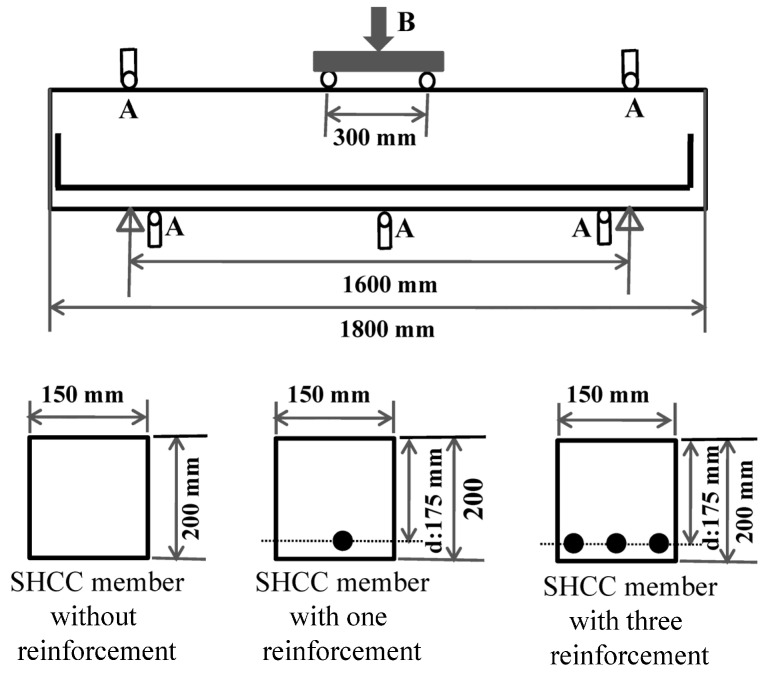
Geometry of experimental specimens during flexural failure.

Figure 3 shows the geometry of the experimental SHCC members subject to flexural failure, which were 1800 mm long with a cross-section of 150 mm × 200 mm. The flexural-failed SHCC member with and without steel reinforcement aimed to investigate the effect of steel reinforcement in improving the crack elongation capacity of the SHCC member. There were three specimens prepared for the experiment; the first SHCC member had no deformed bars in the longitudinal direction, and the other two SHCC members were reinforced with one or three deformed bars (6 mm in diameter) in the longitudinal direction, respectively. The distance between the deformed bars was 50 mm. There are no steel bars employed as stirrups for any of the SHCC members; therefore, the reinforcement ratios of the experiment SHCC members were 0%, 0.42%, and 1.27%, respectively.

The loading of all the SHCC specimens was implemented using a four-point bending test setup in which the points at which displacement was measured are labeled “A” in Figure 3 and the point where the load was measured is labeled “B”. The load was measured using a load cell with a 300 kN gauge capacity and a sensitivity of 0.1 kN, and the transducer used to measure the displacement had a 25 mm gauge range and a sensitivity of 0.002 mm. The transducer required adjustment during the testing process when the measured displacement was greater than its 25 mm gauge range. The experiment was ended when localization among multiple fine cracks was observed.

## 3. Results and Discussion

### 3.1. Behavior of the SHCC Material in the Uniaxial Tension Test

**Figure 4:**
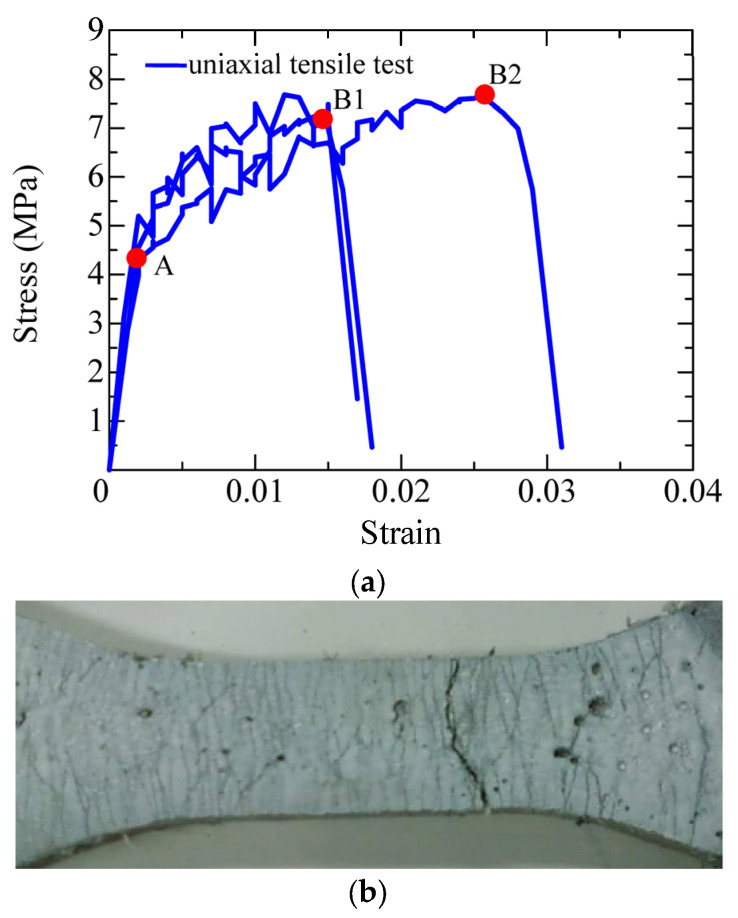
Uniaxial tensile behavior of the SHCC material. (**a**) Uniaxial tensile stress–strain curves; (**b**) Ultimate crack pattern.

**Figure 5:**
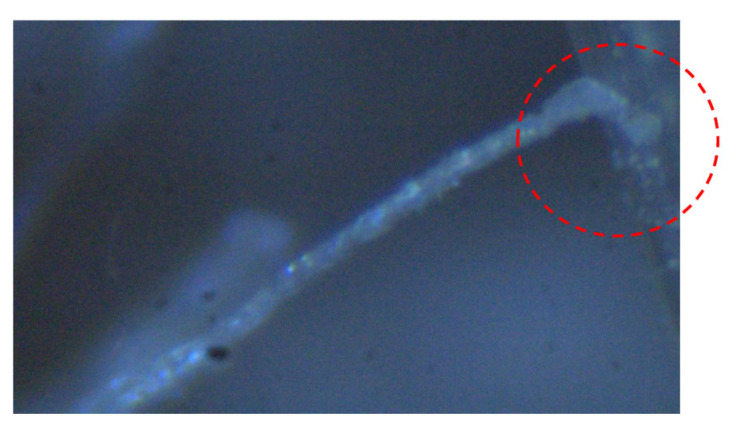
Fiber slipping in the SHCC matrix.

Figure 4 shows the uniaxial tensile behavior of the SHCC material obtained from the uniaxial tension tests using the dumbbell-shaped specimens of small size, including the curves of the tensile stress versus strain and the final distribution pattern of the multiple fine cracks. It can be clearly seen that all the tested specimens displayed excellent strain-hardening performance up to the peak tensile stress. The initial tensile strength of the SHCC material in all the specimens was 4.2 MPa (labeled “A” in Figure 4), which corresponds to the initial cracks appearing in the SHCC specimens. However, variable ultimate tensile strengths of the SHCC material in the specimens occurred at 7 MPa and 8 MPa, respectively (“B1” and “B2” in Figure 4), which corresponds to the hardening strain at 1.5% and 2.2%. This strain-hardening performance of the SHCC material originated from the multiple fine cracks that occurred and propagated after the initial tensile strength, with an initial crack. This was dominated by the fiber bridging effect of the matching between the fibers and the matrix of the SHCC material, in which a slipping phenomenon of the fibers was observed, as demonstrated in Figure 5. Notably, the final failure of the tested specimens was accompanied by localized multiple fine cracks, when the tensile stress of the SHCC material decreased in the uniaxial tensile stress–strain curves.

**Figure 6:**
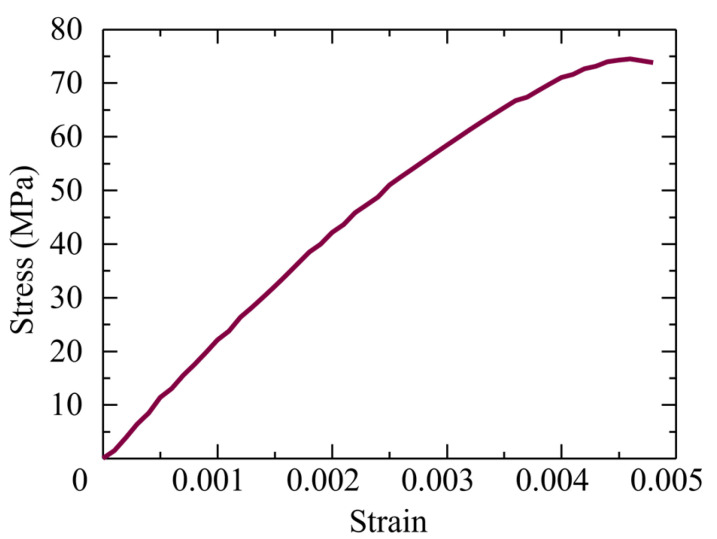
Uniaxial compressive stress–strain graph of the SHCC material.

Figure 6 demonstrates the curve of the compression stress versus strain for the SHCC material during the uniaxial compression test, in which the cylindrical specimens had a 50 mm diameter and a 100 mm vertical length, and the strain gauges were glued to gauge the compression strain. The compression strength and elastic modulus of the SHCC were 72 MPa and 25 GPa, respectively. The elastic modulus and yield strength of the deformed steel bar were 200 GPa and 295 MPa, respectively.

### 3.2. Ductility of the Shear-Failed SHCC Member Compared to That of the SHCC Material under Uniaxial Tension

**Figure 7:**
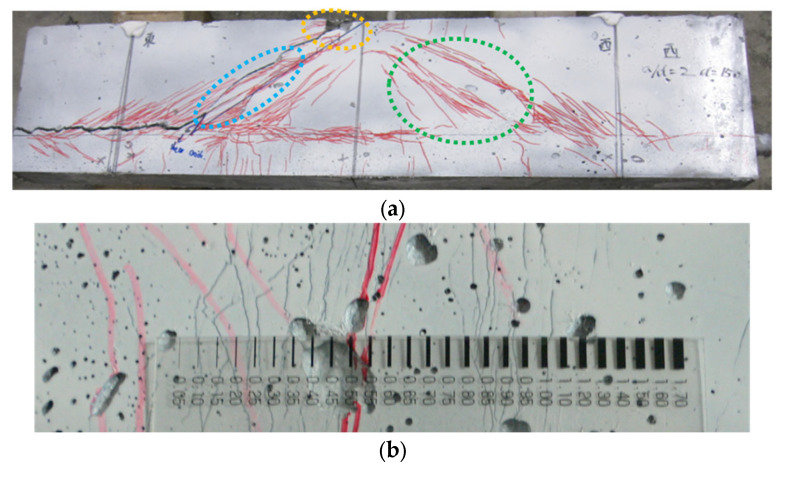

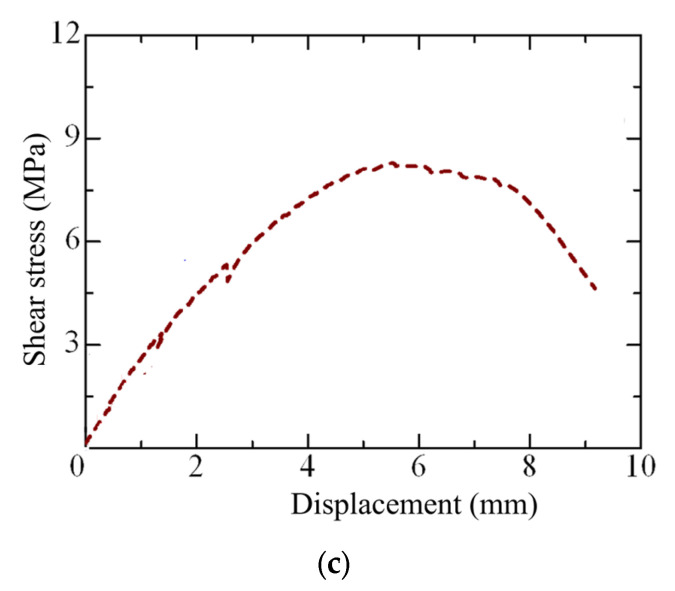
Behavior of the shear-failed SHCC member. (**a**) Distribution of multiple fine cracks in the shear-failed SHCC member; (**b**) Crack spacing of the multiple fine cracks measured in the middle of the shear span; (**c**) Shear displacement curve.

Figure 7 shows the behavior of the shear-failed SHCC member with the expected diagonal shear failure at the end of the shear failure test, including the crack pattern and shear stress displacement curve. It can be confirmed that the crack pattern of the shear-failed SHCC member significantly varied from that of the concrete member; the shear-failed SHCC specimen failed via a diagonal shear, and there were multiple fine cracks in the directions of this diagonal shear (green lines in Figure 7). Some of these multiple fine cracks were localized along the diagonal shear direction (blue lines), and a collapse phenomenon was observed near the loading plate (yellow lines) in the SHCC member with shear failure. The aforementioned multiple fine cracks were distributed in a limited range along the diagonal shear direction of the shear-failed SHCC member, whereas those of the SHCC material subject to the uniaxial tension test and using small-sized specimens were almost evenly distributed within the whole specimen. The crack spacing of the multiple fine cracks in the middle shear span of the SHCC member with shear failure was measured as illustrated in Figure 7b, yielding a value of 3 mm, which is similar to the crack spacing of the multiple fine cracks measured in the dumbbell-shaped SHCC specimens subject to the uniaxial tension test. This implies that the aforementioned limited range in the direction of the diagonal shear in the SHCC member under shear failure retained the multi-cracking performance of the SHCC material in the uniaxial tension tests.

Figure 7c illustrates the curve of the shear stress versus displacement for the SHCC member with shear failure, in which the shear stress was calculated by dividing the effective section area by the loading force. The effective section area is the product of multiplying the section width by the effective height. The shear stress displacement curve of the SHCC member illustrates a linear increase in the shear stress up to the peak value followed by a sudden drop in the shear force post-peak. This was probably induced by the decreased contact effect resulting from the relative smoothness of the cracking surface in the SHCC member compared to the notable contact effect on the surface of the crack in the normal concrete member.

### 3.3. Ductility of the Flexural-Failed SHCC Member without Steel Reinforcement Compared to That of the SHCC Material under Uniaxial Tension

**Figure 8:**
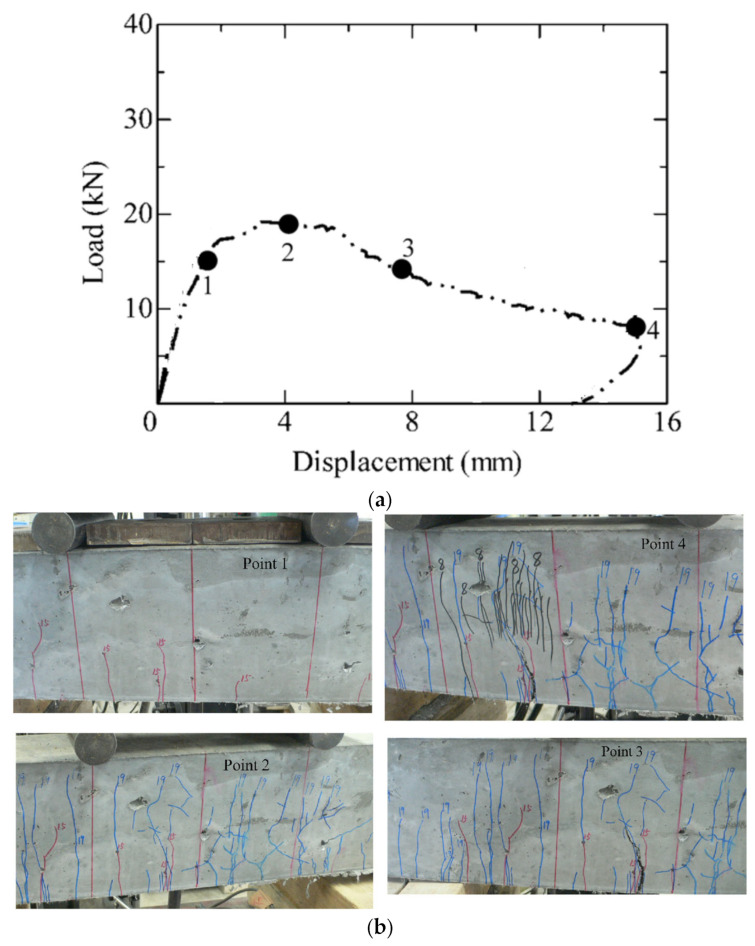
Cracking behavior of specimen F-1, a flexural-failed SHCC member without steel reinforcement. (**a**) Load-displacement curve; (**b**) Multi-cracking behavior.

Figure 8 demonstrates the flexural behavior of the un-reinforced SHCC member in specimen F-1 with a 0% reinforcement ratio, in which the cracking patterns are labeled in the load-displacement curve shown in Figure 8a. It is clear that multiple, flexural fine cracks occurred in this specimen (point “1” in Figure 8a) and increased remarkably with the enhanced load-bearing capacity (point “2”). This implies the flexural-failed SHCC member, specimen F-1, also experienced multi-cracking processes, and the final failure was subsequently accompanied by multiple and localized flexural fine cracks (points “3” and “4”).

### 3.4. Behavior of the Flexural-Failed SHCC Member with Steel Reinforcement

#### 3.4.1. Load-Displacement Curves 

**Figure 9:**
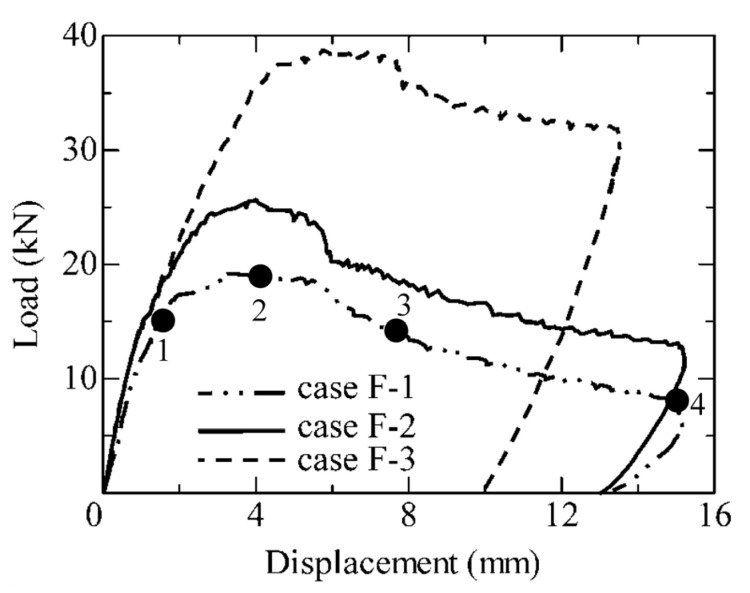
Load-displacement curves of the flexural-failed SHCC members with and without steel reinforcement.

**Figure 10:**
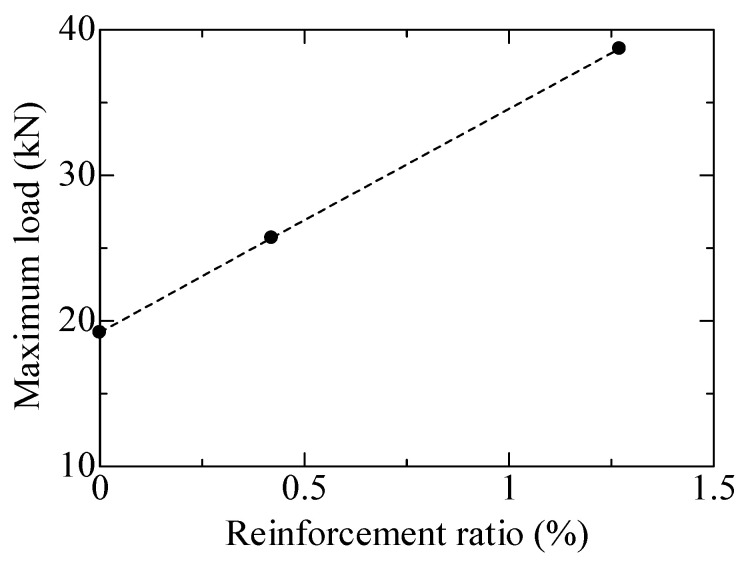
Load-carrying capacity versus the reinforcement ratio of the flexural-failed SHCC members with and without steel reinforcement.

Figure 9 shows the experimentally obtained load-displacement curves for the flexural-failed SHCC members with steel reinforcement, in which the specimens labeled F-1, F-2, and F-3 denote members with a 0%, 0.42%, and 1.27% reinforcement ratio, respectively. It is quite obvious that the load-carrying capacity and deflection of the flexural-failed SHCC members were remarkably raised with the enhanced reinforcement ratio. Specifically, the load-carrying capacities of specimens F-1, F-2, and F-3 were 19.2 kN, 25.7 kN, and 38.7 kN, respectively. It seems that there was a linear increase in the load-bearing capacity with the increase in the reinforcement ratio in these flexural-failed SHCC members, as illustrated in Figure 10. This behavior was probably caused by the enhanced strain-hardening capacity of the flexural-failed SHCC members with steel reinforcement, which is generated from their improved multi-cracking performance.

#### 3.4.2. Cracking Behavior and Patterns

**Figure 11:**
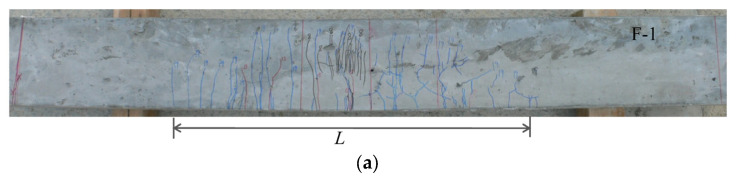

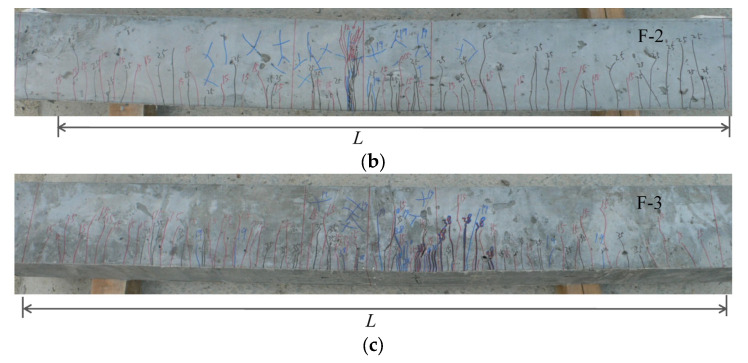
Ultimate crack distribution of the experimental specimens. (**a**) F-1; (**b**) F-2; (**c**) F-3.

Figure 11 illustrates the crack patterns of all the flexural-failed SHCC members with and without steel reinforcement in the experiment. The quantities of the multiple fine cracks *n* in specimens F-1, F-2, and F-3 were 33, 76, and 96, and the distributed lengths *L* in the aforementioned specimens were 810 mm, 1511 mm, and 1600 mm, respectively. This implies that the steel reinforcement effectively increased the crack elongation performance under the multi-cracking phenomenon in these specimens.

The average cracking spacing *Sav* in all the flexural-failed SHCC members can be calculated by dividing the distributed lengths *L* by the quantities of fine cracks *n*. Table 2 lists the average cracking spacings of specimens F-1, F-2, and F-3, which are also compared to the dumbbell-shaped SHCC specimens of small size as used in the uniaxial tension tests. Notably, the ductility reduction of SHCC used for flexural or shear strengthening of reinforced concrete structures also has been reported in the published literature [12,13]. Moreover, the average cracking spacing of the flexural-failed SHCC member with steel reinforcement was effectively reduced with the increasing reinforcement ratio of the members with steel reinforcement. This reflects the fact that steel reinforcement effectively increased the crack elongation performance of the flexural-failed SHCC members, with increasing multi-cracking phenomena.

## 4. Conclusions

In this paper, the ductility variation of flexural-failed and shear-failed SHCC members as well as the ductility improvement of SHCC members with steel reinforcement were experimentally investigated. The study focused on not only the decrease in the crack elongation performance of the SHCC material in structural utilization but also the increase in the crack elongation performance with steel reinforcement. The beneficial effects of SHCC for pre-stressed concrete girder bridges can be experimentally studied in the laboratory in future work. The main conclusions are summarized as follows.

(1) The SHCC material in the uniaxial tension test using dumbbell-shaped specimens of small size exhibited an excellent strain-hardening capacity, with significant multi-cracking performance originating from multiple fine cracks that occurred and propagated following an initial crack. This was dominated by the fiber bridging effect of the matching between the cement matrix and the fibers in the SHCC material, which is significantly influenced by not only the fiber distribution within each section of the SHCC material but also the variation in the fiber content among the different sections.
(2) The crack elongation performance of the shear-failed SHCC member was significantly reduced compared to that of the dumbbell-shaped SHCC specimens of small size subject to the uniaxial tension test. For the shear-failed SHCC member, the aforementioned multiple fine cracks were distributed within a limited range along the direction of the diagonal shear, whereas they were almost evenly distributed across the whole dumbbell-shaped SHCC specimens of small size. However, the measured crack spacing of the multiple fine cracks across the shear span of the shear-failed SHCC member was about 3 mm, and this was similar to that of the dumbbell-shaped SHCC specimens in the uniaxial tension test. This implies that the limited range along the diagonal shear direction of the shear-failed SHCC members retained its multi-cracking performance in the uniaxial tension test.
(3) The multi-cracking performance of the flexural-failed SHCC members was obviously reduced compared to that of the small dumbbell-shaped SHCC specimens in the uniaxial tension test. This illustrates the significant ductility reduction of SHCC in structural utilization since the bridging effect of the short fibers in the flexural-failed SHCC member significantly varied from that of the small dumbbell-shaped specimens under uniaxial tension; the fiber bridging effect in each section of the SHCC specimens correlated with all the nearby fibers and, thus, continuously changed.
(4) The flexural-failed SHCC member without steel reinforcement also experienced multi-cracking processes, with multiple and localized flexural fine cracks appearing in the SHCC member prior to the final failure. However, the multi-cracking performance of the flexural-failed SHCC member without steel reinforcement was significantly reduced compared to that of the SHCC material in the uniaxial tension tests using the dumbbell-shaped specimen of small size. This was probably induced by the aforementioned continuously changing fiber bridging effect in the SHCC member without steel reinforcement.
(5) Steel reinforcement can effectively increase the crack elongation performance with the multi-cracking phenomenon in flexural-failed SHCC members. The average cracking spacing was effectively reduced by an increase in the reinforcement ratio of the flexural-failed SHCC member with steel reinforcement. Moreover, the load-bearing capacity of the flexural-failed SHCC member with steel reinforcement seemed to increase linearly with an increase in the reinforcement ratio. This was probably caused by the enhanced strain-hardening capacity of these members as a result of their improved multi-cracking performance.

## Figures and Tables

**Table 1:** Mix proportions of the SHCC material.

Water/ Binder	Unit Content (kg/m^3^)							
	Water	Cement	Silica Fume	Fine Sand	PE Fiber	Expansion Agent	Superplasticizer	Air Reducing Agent
0.22	338.5	1267.9	230.8	153.9	14.6	40.0	15.4	0.06

**Table 2:** Average cracking spacing *S_av_* of the flexural-failed SHCC members with and without steel reinforcement.

Specimen	*ρ_s_* (%)	*n*	*L* (mm)	*S_av_* (mm)
F-1	0	33	810	24.6
F-2	0.42	76	1511	19.9
F-3	1.27	96	1600	16.7
uniaxial tension	0	38	100	2.6

## Data Availability

Data are contained within the article.
